# Neurocognitive evidence for mental imagery-driven hypoalgesic and hyperalgesic pain regulation

**DOI:** 10.1016/j.neuroimage.2015.07.008

**Published:** 2015-10-15

**Authors:** Francesca Fardo, Micah Allen, Else-Marie Elmholdt Jegindø, Alessandro Angrilli, Andreas Roepstorff

**Affiliations:** a MINDLab, Center of Functionally Integrative Neuroscience, Aarhus University, 8000 Aarhus, Denmark; b Institute of Cognitive Neuroscience, University College London, London WC1N 3AR, United Kingdom; c Wellcome Trust Centre for Neuroimaging, University College London, London WC1N 3BG, United Kingdom; d Interacting Minds Centre, Aarhus University, 8000 Aarhus, Denmark; e Department of General Psychology, Padova University, 35131 Padova, Italy; f CNR Institute of Neuroscience, Padova, Italy

## Abstract

Mental imagery has the potential to influence perception by directly altering sensory, cognitive, and affective brain activity associated with imagined content. While it is well established that mental imagery can both exacerbate and alleviate acute and chronic pain, it is currently unknown how imagery mechanisms regulate pain perception. For example, studies to date have been unable to determine whether imagery effects depend upon a general redirection of attention away from pain or focused attentional mechanisms. To address these issues, we recorded subjective, behavioral and ERP responses using 64-channel EEG while healthy human participants applied a mental imagery strategy to decrease or increase pain sensations. When imagining a glove covering the forearm, participants reported decreased perceived intensity and unpleasantness, classified fewer high-intensity stimuli as painful, and showed a more conservative response bias. In contrast, when imagining a lesion on the forearm, participants reported increased pain intensity and unpleasantness, classified more low-intensity stimuli as painful, and displayed a more liberal response bias. Using a mass-univariate approach, we further showed differential modulation of the N2 potentials across conditions, with inhibition and facilitation respectively increasing and decreasing N2 amplitudes between 122 and 180 ms. Within this time window, source localization associated inhibiting vs. facilitating pain with neural activity in cortical regions involved in cognitive inhibitory control and in the retrieval of semantic information (i.e., right inferior frontal and temporal regions). In contrast, the main sources of neural activity associated with facilitating vs. inhibiting pain were identified in cortical regions typically implicated in salience processing and emotion regulation (i.e., left insular, inferior-middle frontal, supplementary motor and precentral regions). Overall, these findings suggest that the content of a mental image directly alters pain-related decision and evaluative processing to flexibly produce hypoalgesic and hyperalgesic outcomes.

## Highlights

• Specific mental imagery contents bias pain perception towards hypoalgesic and hyperalgesic outcomes
• Mental imagery influences pain-related perceptual decision-making in terms of stimulus discriminability and response bias
• Pain-related N2 amplitudes are modulated by mental-imagery content
• Cortical activity associated with pain inhibition likely reflects cognitive inhibitory control
• Cortical activity associated with pain facilitation likely reflects salience and emotional processing

## Introduction

Mental imagery – the ability to generate internal representations that preserve the core features of a perceptual experience – relies on similar neural mechanisms as those of actual perception (Kosslyn et al., 2001; McNorgan, 2012). This mechanism of shared representation between imagery and perception is common across sensory modalities, including the tactile domain (Gallace, 2013; Olivetti Belardinelli et al., 2009; Schmidt et al., 2014; Yoo et al., 2003). However, the possibility for imagery to engage representations of painful percepts is unknown. Indirect evidence of the interplay between mental imagery and pain perception is provided by clinical investigations of the therapeutic efficacy of motor imagery and spontaneous imagery in chronic pain patients. For example, imagining the movement of an affected limb can temporarily reduce pain symptoms in patients with chronic complex regional pain syndrome and phantom limb pain (e.g., MacIver et al., 2008; Moseley, 2004, 2006). Similarly, clinical investigations showed that patients with chronic pain spontaneously experience distressing mental images that contribute to a negative feedback loop maintaining and exacerbating pain (Berna et al., 2011, 2012; Gosden et al., 2013). The use of coping images has also been found to facilitate control over symptoms (Berna et al., 2011, 2012). However, while the relevance of mental imagery as either a strategy for pain reduction or therapeutic target (as in the case of spontaneous negative images) is well established (Berna et al., 2011), the cognitive and neural mechanisms responsible for imagery-driven modulatory effects on pain perception remain unclear. For instance, it is presently unknown whether pain-related mental images influence perception by merely redirecting attention away from the source of pain towards an internal mental image or rather by acting specifically on sensory or affective pain-related processing. We thus tested whether pain imagery produces specific directional (e.g., hypoalgesic and hyperalgesic) effects, or rather only interferes with pain processing regardless the imagined content.

To address these issues, we assessed the influence of inhibitory and facilitatory mental imagery on pain intensity and unpleasantness using subjective ratings, signal detection measures, pain-related electrophysiological potentials (ERPs) and source reconstruction. Pain subjective ratings provided a measure of the participants' efficacy using imagery to modulate sensory and emotional aspects of pain. The use of signal detection theory allowed us to establish whether the imagery-driven alteration of pain sensations was linked to changes in stimulus discriminability (d-prime) and response bias (criterion) (Macmillan and Creelman, 2004). Further, we applied a mass-univariate approach to identify which of the temporally distinct pain-related ERP components (N2 and/or P2 potentials) reflected the imagery effects in a spatio-temporally unbiased analysis; see Kilner (2013). Finally, we applied a minimum norm source reconstruction method, in order to delineate possible neural origins of the ERP effects associated with the two modulatory conditions.

Specifically, to illuminate the role of mental imagery in modulating pain, we directly contrasted two mechanistic hypotheses. First, we reasoned that if mental imagery primarily depends upon cognitive demand in modulating the upcoming stimuli, we should observe a general attenuating effect of imagery vs. baseline coupled with no difference between pain-inhibitory and facilitatory imagery (“a-directional hypothesis”). In such a case, the effort of generating and maintaining a mental image may serve to distract from or interfere with pain processing, regardless of the imagined content. This hypothesis proposes that hyperalgesic imagery should have a minimal effect on pain exacerbation and that both hypoalgesic and hyperalgesic imagery should correspond to similar amplitudes of pain-related ERPs, as well as similar underlying cortical sources. Alternatively, if mental imagery exerts distinct effects on the processing of stimulus features or affective responses related to upcoming stimuli, we expected content-specific differences when contrasting hypoalgesic and hyperalgesic imagery (“directional hypothesis”). This latter hypothesis would predict similar effects for both hypoalgesic and hyperalgesic effects, albeit in opposite directions, across the subjective, behavioral, scalp and source-level ERP measures. The clarification of this mechanism offers important insights on the role of top-down mental imagery on pain regulation.

## Materials and methods

### Participants

25 healthy volunteers were recruited from Aarhus University and the local community. All participants were proficient Danish speakers, right-handed, with normal or corrected-to-normal vision. No participants reported a history of pain disorders, neurological or psychiatric illness, or use of analgesics. All participants gave informed consent before participation and received a reimbursement of 300 DKK (~ 40EUR). Data from two participants were not included in any analyses due to incomplete data collection (one participant did not complete the experiment, and another had missing behavioral data in most blocks). Two other participants were excluded from statistical analysis on account of excessive EEG artifacts. The final sample included 21 participants (9 female; mean age = 24.5 years; range = 21–36 years). The study was approved by the Ethical Committee of Central Region Denmark and conducted in accordance with the Declaration of Helsinki.

### Task description

**Fig. 1:**
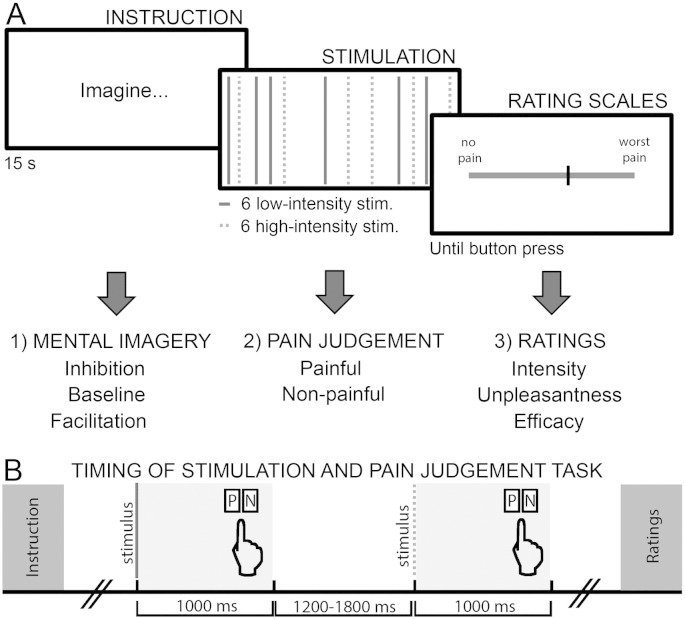
A) Timeline of a single block. Each block started with the presentation of a verbal instruction (1) indicating the upcoming imagery condition. Participants were instructed to use mental images to inhibit, facilitate or experience the stimulation without modulation. The suggested images corresponded to a gloved forearm (inhibition), a wounded forearm (facilitation), or the skin of the forearm (baseline). After 15 s from the instruction onset, 12 (6 high-intensity, 6 low-intensity) stimuli were delivered in a random order. Participants had to judge each stimulus as either “painful” (P) or “non-painful” (N) by pressing a button on the keyboard (2). Following stimulation, participants recalled the worst felt pain to rate their perceived intensity, unpleasantness and efficacy of pain control (3). B) Timeline of stimulation and pain judgment task. Each stimulus lasted for 5 ms. Participants had 1000 ms to judge each stimulus as either painful or non-painful, by pressing a button on the keyboard. After the response, the inter-trial interval varied between 1200 and 1800 ms.

Participants were asked to (1) modulate the upcoming sensory stimulation through script-driven mental imagery, (2) identify each stimulus as either painful or non-painful (pain judgment task), and (3) evaluate their pain experience (pain intensity, pain unpleasantness, and efficacy ratings), see Fig. 1. At the beginning of each block of trials, participants were required to imagine the content of a verbal script and to use the suggested mental images to either inhibit or facilitate the triggered pain responses or to experience pain without modulation (baseline). In the inhibition and facilitation conditions, instructions suggested that participants generate and maintain the mental image of either a glove or a wound on the right forearm to attenuate or to exacerbate pain sensations, respectively. In the baseline condition, instructions were to simply imagine the skin of the right forearm, without any pain modulation. The instruction for inhibition was chosen according to a previous study (De Pascalis et al., 1999), whereas the instruction for facilitation was specifically designed to mirror the inhibition condition, altering the content of the image (wound instead of a glove) and the directionality of the modulation (amplification instead of attenuation). Within each block of trials, participants were required to judge each stimulus as either painful or non-painful as quickly and accurately as possible by pressing two possible keyboard buttons, counterbalanced across participants (Fig. 1). Finally, at the end of each block of trials, participants were invited to rate the worst pain intensity and unpleasantness felt in the previous block and to judge their ability to influence the triggered responses accordingly to the given instruction (efficacy ratings; Fig. 1).

### Procedure

In a single experimental session, participants performed separate pain and empathy-for-pain tasks in a counterbalanced order. The rationale of the empathy-for-pain task was very similar to the pain task explained here, with instructions to either decrease or increase empathy, while viewing pictures of faces with neutral or painful expression. The pain and empathy-for-pain tasks were separated by a pause of 15–20 min. Here, only the pain task is reported. The pain session started with a calibration task identifying the intensity of non-painful and painful stimulation suitable for each participant. Using a bipolar electrode placed on the right forearm over the medial nerve, stimulus trains of increasing intensity (starting intensity = 0.39 mA; step = max 0.39 mA) were delivered via a Digitimer DS5A stimulator (Digitimer, Hertfordshire, UK). Participants rated the intensity of each stimulus on a horizontal visual-analogue scale (VAS; range = 0–10, where 0 equals “no pain sensation”, 1 “just noticeable pain” and 10 “worst imaginable pain”). The calibration terminated when the participant rated intensities with a score greater than 8. Hence, intensities corresponding to 0.8 and 8 ratings on the VAS were chosen for the experimental task. The calibration task was followed by written and oral instructions describing the experimental task and a brief training session consisting of three blocks, one for each condition (i.e., inhibition, baseline, and facilitation). The training always began with the baseline condition, whereas the second block could be either inhibition or facilitation in a counterbalanced order. All participants reported that the three blocks were sufficient for understanding the task.

The experimental task consisted of 24 blocks, 8 for each condition. Each block began with a display of a verbal script informing the participant of the upcoming block condition (i.e., inhibition, baseline, or facilitation; Fig. 1A, “instruction”). Each instruction was presented for 15 s, followed by a 2 s inter-stimulus interval and a random set of 12 stimuli of two fixed intensities (low-intensity and high-intensity; Fig. 1A, “stimulation”). The duration of each stimulus was 5 ms. Importantly, participants were not informed that only two electrical intensities were delivered and were instead instructed that the stimuli could have any intensity corresponding to the range between 0.8 and 8 in the VAS, according to their ratings in the calibration task. Participants were asked to maintain the mental image throughout each block while performing a pain judgment task. After each stimulus, participants pressed a button with either the middle or the index finger of the left hand to indicate whether the stimulus was perceived as painful or not (Fig. 1B). If no button was pressed within 1000 ms of the stimulation, the response was marked as missing. Buttons identifying perception of painful and non-painful stimuli were counterbalanced across participants. The inter-stimulus interval (ISI) corresponded to 1200–1800 ms after participants' response. Following each set of stimuli, participants were asked to recall the most painful stimulus felt and to provide ratings on three VASs (Fig. 1A, “rating scales”). On the first scale, participants rated the highest painful intensity they felt (“How much pain did you feel?”; 0 = no pain, 10 = the worst imaginable pain). On the second scale, they were asked to rate the highest unpleasantness (“How unpleasant did you feel?”; 0 = no unpleasantness, 10 = the worst imaginable unpleasantness). Finally, in inhibition and facilitation blocks only, they were asked to rate their ability to influence their pain experience (“How efficient were you in influencing your sensations?”; 0 = no control, 10 = perfect control). After each rating session, 5-s intervals separated contiguous blocks. The blocks were presented with two possible pseudo-randomized orders (direct or reverse) to counterbalance order effects. The direct order was the following: B, B, F, F, I, I, B, B, I, I, F, F, I, I, F, F, B, B, F, F, I, I, B, and B (where B corresponds to baseline, F to facilitation, I to inhibition).

Before and after the pain and empathy tasks, 5-min resting EEG sessions were recorded, while participants were sitting relaxed on the chair with their eyes open. Between the second and the third session (i.e., in between the two tasks), participants completed a computerized form of the Interpersonal Reactivity Index (Davis, 1980) and a short version of the Tellegen's Absorption Scale (Tellegen and Aktinson, 1974), for an assessment of individual differences in empathy and absorption, namely the ability to be engaged by thoughts, feelings and mental images. Data of the empathy task and IRI are not reported here.

### Indices of subjective pain modulation

We calculated modulatory intensity and unpleasantness indices for each participant to highlight the rate of change (Δ) in terms of points in the VAS scale, by subtracting the mean rating at baseline from the mean rating at modulation (e.g., pain inhibition modulatory index corresponds to ΔI = mean I rating − mean B rating, whereas pain facilitation modulatory index corresponds to ΔF = mean F rating − mean B rating). These indexes were then transformed into percentages. Thus, modulatory indexes estimated the degree to which pain perception varied during each imagery condition.

### Signal detection theory and pain

The participants' performance on the pain judgment task was assessed by signal-theoretic measures (i.e., d-prime and criterion) and reaction times. Signal detection theory (SDT) estimates participants' sensitivity (d-prime or d′) and response bias (criterion) while either detecting the presence of a stimulus or judging stimuli of two different intensities (Macmillan and Creelman, 2004). The measure d′ indicates the degree of stimulus sensitivity or discriminability, whereas the criterion reflects the participants' decision-making in terms of a more conservative or liberal decision bias in responding whether a certain stimulus has occurred. STD was thus used to identify whether changes in performance were driven by either alteration of d′, criterion or a combination of the two as a result of our conditional manipulation.

In the present experiment, d′ and criterion were measured in relation to a binary decision, namely “painful” or “non-painful” responses with respect to high and low-intensity stimuli. Participants' responses were classified on the basis of an ideal performance, with high-intensity stimuli judged as painful and low-intensity stimuli as non-painful. Thus, “hits” corresponded to the classification of high-intensity stimuli as painful; “misses” to high-intensity stimuli classified as non-painful; “false alarms” to low-intensity stimuli classified as painful, and finally “correct rejections” to low-intensity stimuli classified as non-painful. As only two outcomes provide independent information about the participant's performance, d′ and criterion are computed using the proportion of hit (hit rate; H) and false-alarm trials (false alarm rate; F). If a hit and false alarm pair corresponded to perfect performance (i.e., H = 1 and F = 0), we converted F = 0 to F = .01 using the formula 1 / (2N) and H = 1 to H = .99 using the formula 1 − 1 / (2N) (Macmillan and Creelman, 2004).

The sensitivity measure d′ was calculated as the normalized difference between hit and false alarm rates for each instruction (i.e., inhibition, baseline and facilitation). The formula corresponded to d′ = z(H) − z(F). Specifically, d′ measured the ability to categorize the two classes of stimuli across the three different instruction conditions. Higher sensitivity values were obtained when the participant's responses were closer to the ideal performance (high-intensity = “pain”; low-intensity = “no-pain”). Instead, lower sensitivity values corresponded to an increased mismatch between the ideal and the actual classification. Ceiling effect corresponded to d′ = 4.65.

Alternatively, criterion was calculated as the normalized sum of hit and false alarm rates multiplied by − .5 for each instruction (i.e., inhibition, baseline and facilitation). The formula corresponded to c = [z(H) + z(F)] ∗ − .5. The criterion provided information about the participants' response bias in judging the stimuli as painful with a more conservative or liberal bias across conditions. A more conservative bias corresponds to positive criterion values. Instead, a more liberal bias corresponds to negative criterion values. Unbiased responses are obtained when the criterion value is close to zero. As changes in sensitivity (d′) and response bias (criterion) are independent, signal detection theory distinguishes between discriminative and motivational components in perceptual decision-making.

Our application of SDT to pain differed from previous studies; for reviews and also a critique, see Lloyd and Appel (1976) and Rollman (1976, 1977). Here, we used SDT not to identify pain threshold or tolerance, but to determine mental imagery-driven changes in sensitivity and response bias across conditions that were identical in terms of stimulus intensities (50% low-intensity; 50% high-intensity stimuli). Importantly, the two levels of intensity were calibrated for each participant to reflect non-painful and painful perception in a neutral condition before the beginning of the experimental task.

### Reaction times and pain

Reaction times measured the participants' speed in judging the high and low-intensity stimuli. Average reaction times (RTs) were calculated for each stimulus intensity and instruction condition, excluding false alarms (low-intensity stimuli judged as painful) and misses (high-intensity stimuli judged as non-painful). This exclusion was based on the assumption that response times for hits and correct rejections (matching trials) vs. false alarms and misses (non-matching trials) might reflect different underlying cognitive processes and as such the inclusion of all trials is likely to prevent correct identification of which conditions are driving our experimental effects of interest. Hence, we focused only on the neural correlates of matching trials both to ensure reliable conditional estimates and to facilitate a clear interpretation of our results. The average RTs for miss and false alarm trials were not analyzed given the low overall rate of these trials (average ± SE: false alarms = 5.57 ± 1.03 trials; misses = 7.81 ± 1.04 trials).

### Statistical analysis on subjective and behavioral data

Subjective ratings of perceived pain intensity and unpleasantness, as well as signal detection measures (d′ and c) were analyzed using one-way repeated measures ANOVAs, with the within-subject factor instruction (3 levels: inhibition, baseline, facilitation). Reaction times were analyzed using two-way repeated measures ANOVAs. The two within-subject factors were stimulus intensity (2 levels: high, low) and instruction (3 levels: inhibition, baseline, facilitation). Statistical significance was set at p < .05 and effect sizes were calculated using the partial η_2_. When the assumption of sphericity was violated, the p-values were adjusted according to the Huynh–Feldt correction (HF ε), and in case of significant effects, the Tukey HSD test was applied for post-hoc comparisons.

Paired sample t-tests were applied to determine mean differences in subjective efficacy ratings, as well as intensity and unpleasantness indices across inhibitory and facilitatory conditions. Finally, to investigate factors driving pain control, we estimated a Pearson's correlation exploring the relationship of inhibition and facilitation efficacy ratings. All subjective and behavioral analyses were performed using the data analysis software system STATISTICA (version 8.0, www.statsoft.com).

### EEG recording

The E-Prime v.2.0 (PST, Inc.) software package was used for instructions, stimulation and presentation of VASs. Continuous EEG data were recorded with a 64-electrode active cap (actiCAP) and amplified (BrainAmp MR plus amplifiers) using the Brain Vision Recorder software (Brain Products, Munich, Germany). Two electrodes (i.e., PO9 and PO10) were removed from the cap and placed on the superior orbit and on the outer canthus of the right eye to detect vertical and horizontal eyes movements. The EEG was referenced to the FCz electrode, grounded at AFz, and sampled at 1000 Hz. The impedance was kept below 25 kΩ.

### EEG data analysis

#### Preprocessing

EEG data were processed using EEGLAB (Delorme and Makeig, 2004) and SPM8 (Statistical Parametric Mapping 8, http://www.fil.ion.ucl.ac.uk/spm). Using EEGLAB, the continuous EEG was downsampled to 500 Hz, band-pass filtered (0.1–30 Hz) and segmented into 600-ms stimulus time-locked epochs (− 100/+ 500 ms). An independent component analysis (ICA) algorithm was applied to identify, select, and discard components representative of eye movements and an electrical artifact induced by the stimulation. The segments were then baseline-corrected using the average pre-stimulus activity (− 100/0 ms). Data were re-referenced to the algebraic mean of the left and right mastoids for scalp analysis of the N2 and P2 potentials and to the mean of all electrodes (i.e., average reference) for source reconstruction of the ERP effects. The following steps were identical for both datasets. Then, the two electrodes used to detect eye movements were removed from subsequent processing and the original reference activity (FCz) was reconstructed. Following downsampling and ICA, single-trial epoched data corresponding to high-intensity stimuli judged as painful and low-intensity stimuli classified as non-painful were imported to SPM8 for final preprocessing and statistical analysis. The exclusion of epochs corresponding to stimulus intensities deviating from ideal performance (i.e., false alarms and misses) resulted in 14% of the data being discarded. The rationale for the exclusion was the same previously applied for RTs. Further, epochs with extreme values exceeding a threshold of 150 mV were automatically detected, leading to an additional 1.8% of data discarded. Moreover, bad channels were visually identified, marked for removal and then interpolated. Robust averaging was applied for further artifact removal (Wager et al., 2005). This averaging method down-weights the contribution of outliers, canceling out extreme values, e.g., the portion of signal contaminated by artifacts, providing a good alternative to classical averaging, if artifacts do not regularly occur at the same time points across trials. A visual inspection of the data confirmed that this assumption was not violated. Thus, epochs were averaged separately for each condition, leading to six average waveforms corresponding to high-intensity inhibition, high-intensity baseline, high-intensity facilitation, low-intensity inhibition, low-intensity baseline, and low-intensity facilitation. To perform statistical analysis on ERPs across subjects, we converted the ERP amplitudes for each condition into three-dimensional scalp maps including two-dimensional sensor-space (x, y) and time (z) (Kilner and Friston, 2010; Litvak et al., 2011). For each participant, each time point of the averaged conditions was transformed into a two dimensional 64 × 64 pixel scalp map using linear interpolation and concatenated over the interval from 0 to 500 ms. The obtained 3D scalp map volumes (i.e., 6 images for each participant) were smoothed with a low-pass kernel (6 mm × 6 mm × 6 ms; full width at half maximum, FWHM) and entered into a general linear model (GLM) analysis. The time interval of interest for the statistical analysis did not include the baseline (− 100/0 ms), which by definition cannot differ across conditions and across subjects.

#### Source reconstruction

Averaged referenced data were used for source reconstruction. As individual MRIs were not available, we used a template brain. A three-shell sphere (skin–skull–brain) was used to compute independent identically distributed (IID) solutions, as implemented in SPM8 (Litvak et al., 2011). This inversion method, equivalent to classical minimum-norm, determines the combination of sources that have the minimum total power (Hämäläinen and Ilmoniemi, 1994). The choice of the method was grounded on the assumptions of few distributed neural sources underlying the ERP effects. Further, compared with dipole-based methods, minimum norm estimates do not need a priori hypotheses regarding the distribution of active sources. The time window was restricted to the period in which significant instruction effects were present as revealed by scalp-level statistics. As for the scalp map volumes, the 3D current density maps were smoothed with a low-pass kernel (6 mm × 6 mm × 6 ms; full width at half maximum, FWHM) and entered into a general linear model (GLM) analysis.

#### Statistical analysis of scalp and source maps

We first conducted a general linear model (GLM) analysis of stimulus intensity by instruction interactions, as well as instruction and intensity main effects, across temporal and spatial dimensions on scalp maps. Each individual conditional average was modeled in a flexible factorial design with the factors subject (21 levels), stimulus intensity (2 levels: high, low), and instruction (3 levels: inhibition, baseline, facilitation). The analysis was performed on the time window from 0 to 500 ms, to capture two well-known pain-related ERPs, namely the N2 (around 100–200 ms) and P2 potentials (around 200–400 ms) (Bromm and Lorenz, 1998). Uni-directional t-contrasts were used to evaluate the overall effect of stimulus intensity (high and low), and our two alternative hypotheses (a-directional and directional). To assess differences in amplitudes related to stimulus intensity, we conducted paired t-contrasts for the stimulus intensity main effect, collapsing the three instruction conditions (high > or < low). Second, to test the a-directional hypothesis, we contrasted the modulation conditions inhibition and facilitation vs. baseline (inhibition + facilitation > or < baseline). Finally, to address the directional hypothesis, we contrasted inhibition vs. facilitation, i.e., tested for responses driven by their specific modulatory content (inhibition > or < facilitation); and each imagery condition vs. baseline (inhibition > or < baseline; facilitation > or < baseline). Significance thresholds were corrected for multiple comparisons, across sensors and time points, using Gaussian random field theory to adjust the threshold for null hypothesis rejection (Kilner and Friston, 2010; Litvak et al., 2011). The statistical parametric maps were thresholded at p_UNC_ < .001 (peak-level, uncorrected) and corrected for multiple comparisons based on cluster size using a family-wise error rate set at p_FWE_ < .05.

Mass univariate statistics, in conjunction with appropriate multiple comparison corrections, enable identification of conditional differences across electrodes and time windows, avoiding the use of a priori spatial or temporal regions of interest which may inflate the risk of false positive (type-II) errors when used inappropriately (Crowley et al., 2012; Lage-Castellanos et al., 2010). This approach reduces the risk of biasing statistical results, and offers the opportunity to maximize the information obtained when recording from a large electrodes array. Indeed, if analyses are guided by a posteriori observations of where or when the effects appear, type-II errors rates can be grossly inflated (Kilner, 2013; Kriegeskorte et al., 2009). On the other hand, if time windows and electrodes are a-priori selected on the basis of previous studies, it is possible that important effects may remain undiscovered. Thus, the mass-univariate approach provides a balance of sensitivity and specificity in large-array EEG investigations.

We then conducted a general linear model (GLM) analysis of the instruction main effects at the source level. As for the scalp maps, each individual conditional average was modeled in a flexible factorial design with the factors subject (21 levels), stimulus intensity (2 levels: high, low), and instruction (3 levels: inhibition, baseline, facilitation). The analysis was performed on the time window from 122 to 180 ms, as this period was associated with significant instruction effects at the scalp level. Uni-directional t-contrasts were used to evaluate the overall effect of inhibition vs. facilitation, inhibition vs. baseline, facilitation vs. baseline, and modulation (inhibition and facilitation) vs. baseline. Significant effects were thresholded at p_FWE_ < .05 corrected for both peak- and cluster-level, with a minimum cluster size of 10 voxels. Labels for each significant source location were determined using cytoarchitectonic probabilistic maps from SPM Anatomy Toolbox (Eickhoff et al., 2005).

## Results

### Subjective ratings

**Fig. 2:**
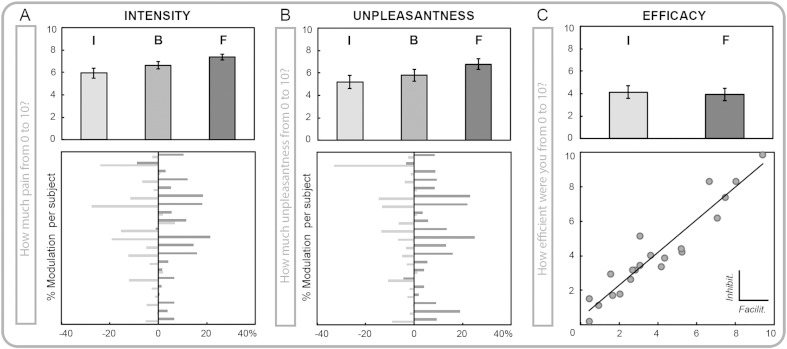
Subjective ratings for the inhibition (I), baseline (B) and facilitation (F) conditions. A) Mean and SE of pain intensity ratings (top panel) and corresponding inhibition and facilitation modulatory index for each participant (bottom panel). The index corresponds to the difference of each modulation condition minus baseline (I–B and F–B) in % modulation. B) Mean and SE of pain unpleasantness ratings (top panel) and corresponding inhibition and facilitation modulatory index for each participant (bottom panel). The index corresponds to the difference of each modulation condition minus baseline (I–B and F–B) in percentage. C) Mean and SE of the perceived efficacy in pain modulation (top panel) and Pearson's correlations between the perceived efficacy under inhibition and facilitation (r = .95; bottom panel).

Participants reported significant differences in the perceived pain intensity and unpleasantness experience according to the given instruction. Compared to baseline, inhibition and facilitation blocks were associated with significantly decreased or increased intensity and unpleasantness ratings, respectively. The instruction main effect for subjective intensity ratings corresponded to F_(2,40)_ = 22.29, partial ƞ_2_ = 0.52, p < .001 (Fig. 2A). Further, the instruction main effect for subjective unpleasantness ratings was F_(2,40)_ = 32.61, partial ƞ_2_ = 0.62, p < .001 (Fig. 2B). To clarify the extent to which participants reported effective modulation of intensity and unpleasantness, we calculated four modulatory indices corresponding to the difference between the ratings under each instruction condition minus the corresponding rating at baseline. The four resulting indices were ΔI Intensity, ΔF Intensity, ΔI Unpleasantness, ΔF Unpleasantness. The intensity modulation indexes showed considerable variability across participants, ranging from no modulation at all to decrements of 27% (mean ± SE = 6.92 ± 1.93%) and increments of 21% (mean ± SE = 7.52 ± 1.62%). Similarly, the unpleasantness modulation indexes varied from no modulation at all to decrements of 33% (mean ± SE = 6.1 ± 1.71%) and increments of 25% in the ratings (mean ± SE = 9.64 ± 1.74%). The inhibition vs. facilitation modulation indexes did not differ for both the intensity (t_(20)_ = 1.03, p = n.s.) and the unpleasantness ratings (t_(20)_ = − 1.39, p = n.s.). The mean value of intensity and unpleasantness modulation for each participant is represented in Figs. 2A and B.

Crucially, participants were asked to rate the degree to which they were able to modulate pain sensations in either direction after each block. The results showed that the perceived efficacy ratings for pain inhibition (mean rating ± SE = 4.13 ± 0.56) and pain facilitation (mean rating ± SE = 3.93 ± 0.56) did not differ (t_(20)_ = 1.09, p = n.s.); participants reported feeling equally capable of inhibiting or facilitating pain. The mean values of the efficacy ratings for each participant are represented in Fig. 2C. Interestingly, the efficacy ratings under inhibition and facilitation were strongly correlated (r = .95, p < .001; Fig. 2C).

### Signal detection theory

**Fig. 3:**
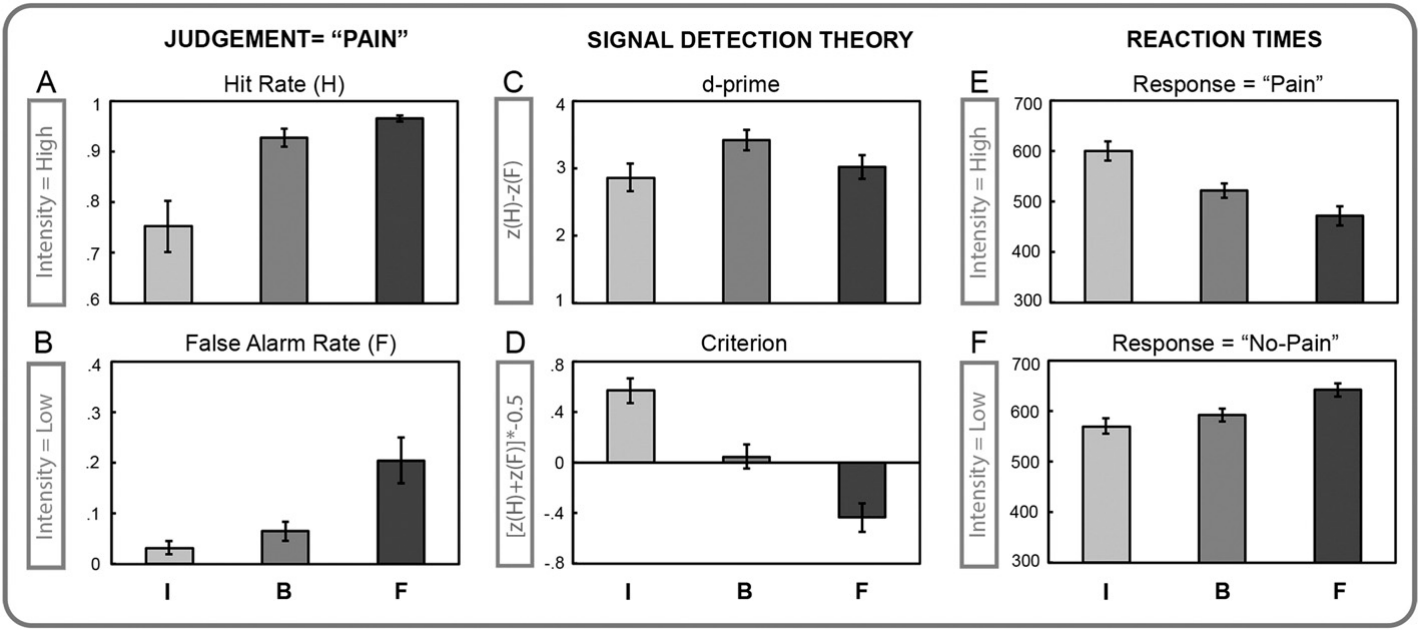
Behavioral results for the inhibition (I), baseline (B) and facilitation (F) conditions. Mean and SE of A) hit rate (high-intensity stimuli judged as painful); B) false alarm rate (low-intensity stimuli judged as painful); C) signal-theoretic d-prime indexing stimulus discriminability; D) decision criterion indexing response bias; E) average reaction times associated with judgment of high intensity stimuli as painful; F) average reaction times associated with judgment of low intensity stimuli as non-painful.

The behavioral performance in the pain judgment task was evaluated in terms of the signal theoretic measures d′ and criterion, which are two measures depending upon the rate of hits or correct responses (i.e., high-intensity stimuli classified as painful) and false alarms (i.e., low-intensity stimuli classified as painful). Two separate ANOVAs performed on the hit and false-alarm rates showed instruction main effects for both measures (hit rate: F_(2,40)_ = 15.34, partial ƞ_2_ = 0.43, p < .001, Fig. 3A; false-alarm rate: F_(2,40)_ = 14.00, partial ƞ_2_ = 0.41, p < .001, Fig. 3B). With respect to baseline, the hit rate was significantly lower when inhibiting pain, while the false alarm rate was significantly higher when facilitating pain. During inhibition, 36.14 ± 2.46 high-intensity trials and 1.52 ± 0.64 low-intensity trials were classified as painful. During baseline, 44.62 ± 0.87 high-intensity trials and 3.14 ± 0.93 low-intensity trials were classified as painful. Finally, during facilitation, 46.52 ± 0.35 high-intensity trials and 9.81 ± 2.16 low-intensity trials were classified as painful.

The sensitivity measure d′ described the participants' performance in classifying high and low-intensity stimuli as “painful” and “non-painful” during the three different instruction conditions. The ANOVA on d′ showed a significant instruction main effect (d′: F_(2,40)_ = 5.70, partial ƞ_2_ = 0.22, p < .01; Fig. 3C). These results demonstrated that participants judged the two classes of stimuli differently, in accordance with the given instruction. The average participants' response during baseline was closer to the ideal performance with respect to inhibition (B > I, p < .01) and facilitation (B > F, p = .06). No difference in d′ was found for the contrast inhibition vs. facilitation (I > F, p = .62). This null effect was due to a similar difference between hit and false alarms rates within the two instruction conditions, although hit and false alarm rates significantly differed when inhibiting and facilitating pain.

Further, response criterion (c) represented a measure of how biased participants were in judging the stimuli as painful. The ANOVA on c found a significant instruction main effect (F_(2,40)_ = 32.76, partial ƞ_2_ = .62, p < .001; Fig. 3D). Participants showed unbiased judgment at baseline, but a more conservative bias during inhibition (I > B, p < .001) and a more liberal bias during facilitation (B > F, p = .001). Participants were less willing to report pain when instructed to inhibit the upcoming sensations, whereas they more willing to report painful sensations following the facilitation instruction.

### Reaction times

In the pain judgment task, behavioral performance was evaluated in terms of how quickly participants classified high and low-intensity stimuli as painful and non-painful in the three different conditions. The ANOVA on RTs showed significant stimulus intensity by instruction interaction (F_(2,40)_ = 39.25, partial ƞ_2_ = 0.66, HF ε = .63, p < .001; Figs. 3E and F), stimulus intensity main effect (F_(1,20)_ = 35.63, partial ƞ_2_ = 0.64, p < .001) and instruction main effect (RTs: F_(2,40)_ = 7.29, partial ƞ_2_ = 0.27, p < .01). The results revealed a differential modulation of participants' speed in judging the stimuli as either painful or non-painful, in accordance with the given instruction. Indeed, participants classified high-intensity stimuli as painful with slower RTs under inhibition (p < .001) and faster RTs under facilitation (p < .05), as compared to pain baseline. The opposite pattern was found for low-intensity stimuli, where participants judged them as non-painful with slower RTs under facilitation (p < .05), compared to the no-pain baseline. No statistical between-condition differences emerged for inhibition vs. baseline when categorizing low-intensity stimuli.

The stimulus intensity main effect indicated that overall, high-intensity stimuli were detected faster than low-intensity ones. Accordingly, post-hoc comparisons confirmed that participants were significantly faster in responding to high vs. low-intensity stimuli at baseline (p < .001, mean difference of 70 ± 55 ms) and under facilitation (p < .001, mean difference of 170 ± 91 ms). In contrast, participants responded similarly to the two levels of stimulus intensity under inhibition (mean difference of − 29 ± 90 ms, p = n.s.). The accelerating effect of high vs. low-intensity observed in the baseline condition was thus greatly increased during facilitation, but abolished during inhibition. Stimulus intensity-related information was thus differentially conveyed to the motor system in accordance with the instruction condition.

### Mental Imagery Assessment

According to the evaluations at the 12-items Tellegen's Absorption Scale (TAS), the participants can be considered ‘average imagers’ (mean ± SE = 6.76 ± 0.39, min = 3, max = 9). To explore the relationship between absorption and efficacy in pain modulation, we carried out a regression analysis with TAS scores as predictor and the efficacy ratings (associated with inhibition and facilitation) as dependent variables. The TAS scores failed to predict both the efficacy ratings of pain inhibition and facilitation (r = .05, r = .01, respectively).

### ERP results

#### Replication of canonical stimulus intensity effects on N2 and P2 potentials

**Fig. 4:**
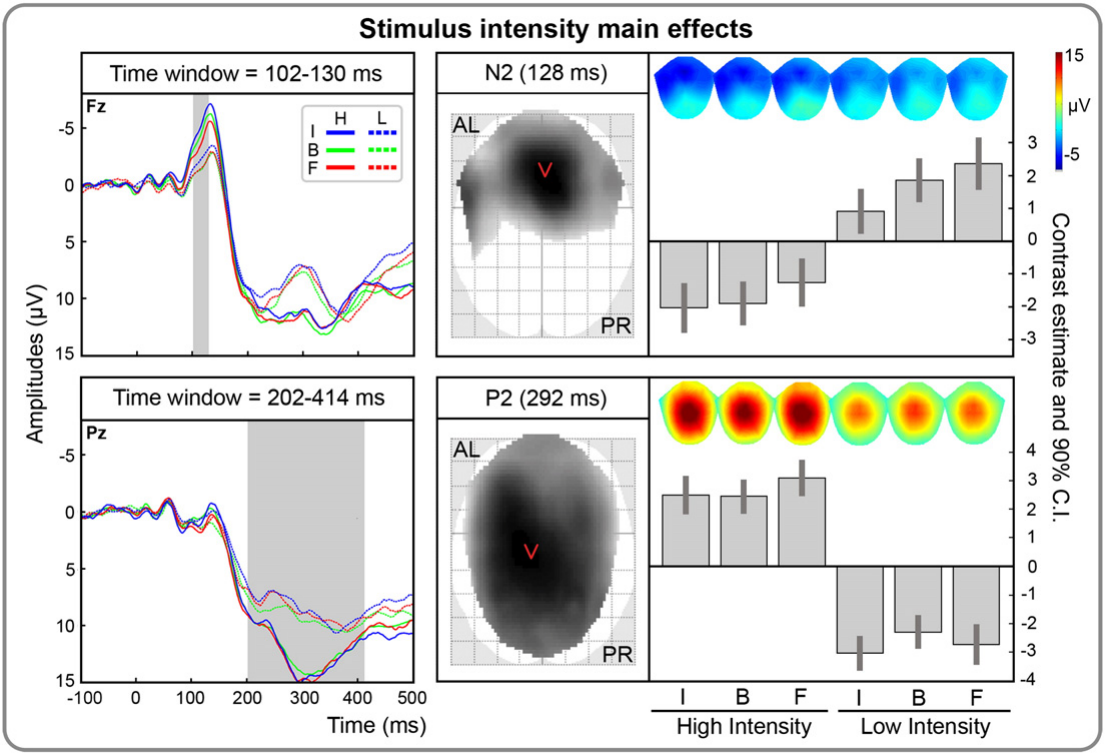
Temporal, spatial and effect size information associated with the stimulus intensity main effects at the scalp-level. First column. Grand-mean ERPs, time locked to the stimulation, separately for painful (full line) and non-painful stimuli (dotted line) and for instruction (blue = inhibition, I; green = baseline, B; red = facilitation, F). Time scale is from − 100 to 500 ms. Negativity is displayed upward. The gray areas represent the temporal extent of significant stimulus intensity main effects at the cluster level (p_FWE_ < .05 cluster-level; p_UNC_ < .001 peak-level). The significant time-windows are overlaid in gray on a representative channel. In the top panel, the significant stimulus intensity main effect between 102 and 130 ms, corresponding to increased N2 amplitudes for high vs. low stimulus intensity, is displayed over Fz. In the bottom panel, the significant stimulus intensity effect between 202 and 414 ms, corresponding to increased P2 amplitudes for high vs. low stimulus intensity, is depicted over Pz. Second column. Statistical parametric maps overlaid over the glass brain showing the spatial extent of the significant effects at 128 and 292 ms (i.e., when maximally significant). Topographical maps, as well as contrast estimates and 90% confidence intervals are depicted for the maximally significant effects at 128 ms and 292 ms for high and low intensity stimuli and for each instruction (inhibition, I; baseline, B; facilitation, F). AL = anterior left; PR = posterior right.

Stimulus intensity main effects were found at 102–130 ms post-stimulus, over an anterior-central cluster (peak-level T_max_ = 5.88; cluster-level p_FWE_ < .001; Fig. 4 and Table 1). This effect corresponded to increased N2 amplitudes elicited by high vs. low-intensity stimuli. Additional stimulus intensity main effects, corresponding to increased P2 amplitudes elicited by high vs. low-intensity stimuli, were found in a central cluster at 202–414 ms (peak-level T_max_ = 10.56; cluster-level p_FWE_ < .001; Fig. 4 and Table 1). These results show that the classic pain-related N2 and P2 components were distinctly modulated by stimulus intensity.

#### A-directional hypothesis

To assess whether ERP pain-related responses are influenced by hypoalgesic and hyperalgesic mental imagery in terms of similar mechanisms, we collapsed the two modulation conditions and compared them to baseline. Neither the intensity by instruction interaction, nor the instruction main effect revealed any result surviving FWE cluster corrections.

#### Directional hypothesis

**Fig. 5:**
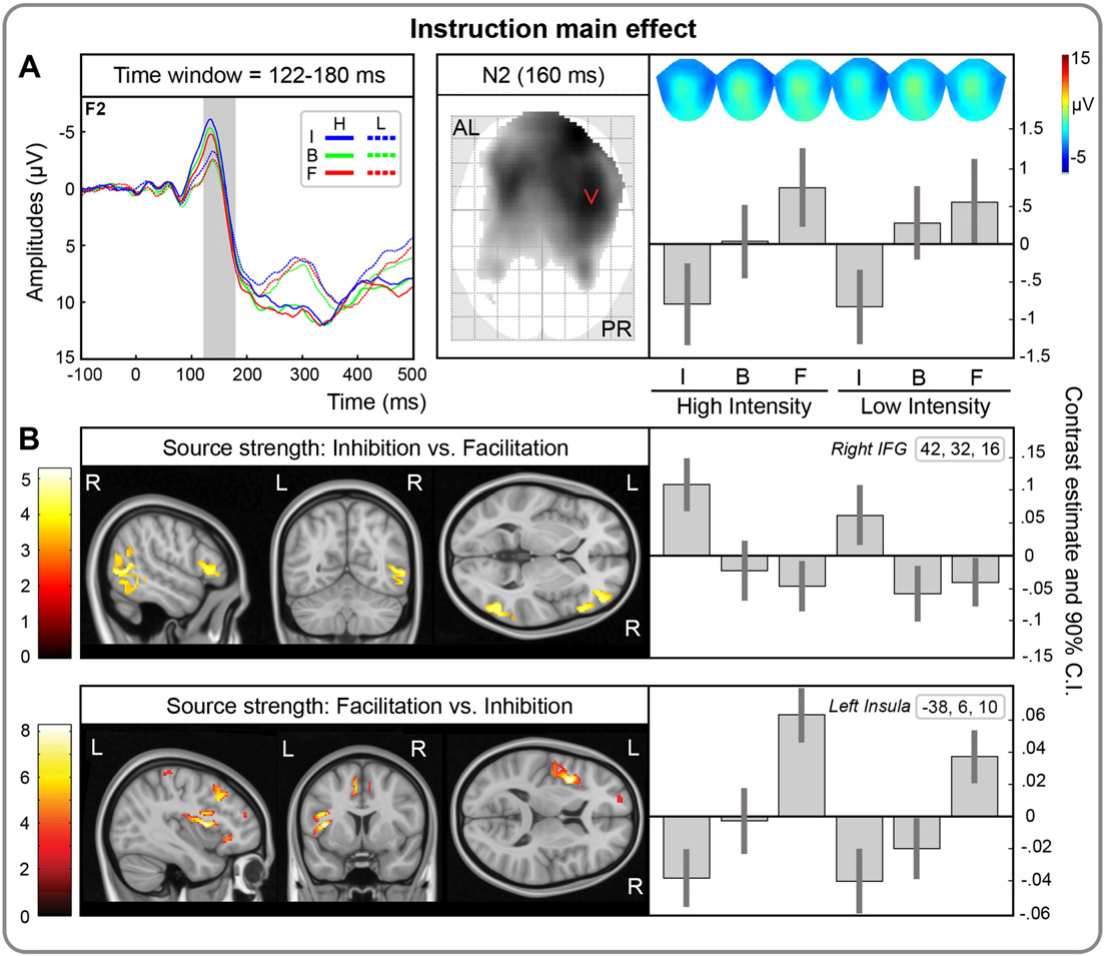
Temporal, spatial and effect size information associated with the instruction main effect at the scalp and source level. A) Top-left: grand-mean ERPs, time locked to the stimulation, separately for painful (full line) and non-painful stimuli (dotted line) and for instruction (inhibition, I; baseline, B; facilitation, F). Time scale is from − 100 to 500 ms. Negativity is displayed upward. The gray area represents the temporal extent of the significant instruction main effect at the cluster level (p_FWE_ < .05 cluster-level; p_UNC_ < .001 peak-level). The significant difference, corresponding to increased N2 amplitudes for inhibition vs. facilitation between 122 and 180 ms, is overlaid on a representative channel, i.e., F2. Top-right: Statistical parametric maps overlaid over the glass brain showing the spatial extent of the significant effect at 160 ms (i.e., when maximally significant). Topographical maps, as well as contrast estimates and 90% confidence intervals are depicted for the maximally significant effects at 160 ms for high and low intensity stimuli and for each instruction (inhibition, I; baseline, B; facilitation, F). AL = anterior left; PR = posterior right. B) Differences in source strength between inhibition vs. facilitation (middle panel) and facilitation vs. inhibition (bottom panel), as well as contrast estimates and 90% confidence intervals for the maximally significant effects in right inferior frontal gyrus (coordinates = [42, 32, 16]) and left insular cortex (coordinates = [− 38, 6, 10]), respectively. The source maps are thresholded at p_UNC_ < .001 for visualization.

To evaluate whether ERP amplitudes are specifically modulated by the content of the mental imagery, we compared inhibition vs. facilitation conditions with two unidirectional t contrasts (I > F and I < F). No significant intensity × instruction interaction was found. However, a significant instruction main effect was observed at 122–180 ms for the contrast I < F over an anterior right cluster (Fig. 5 and Table 2). The stimuli elicited greater negative amplitudes under inhibition compared to facilitation, regardless of stimulus intensity (peak-level T_max_ = 5.00; cluster-level p_FWE_ < .001; Fig. 5 and Table 2). We thus performed planned follow-up contrasts to determine whether either condition diverged from baseline; i.e., I < B and B < F. In the inhibition vs. baseline follow-up contrast, a significant instruction effects survived cluster correction at 156–168 ms (peak-level T_max_ = 4.21; cluster-level p_FWE_ = .003; Fig. 5 and Table 2). Finally, in the facilitation vs. baseline follow-up no significant intensity by instruction interaction or instruction main effect survived the cluster correction. However, cluster-level uncorrected results revealed an instruction main effect at 160–162 ms (peak-level T_max_ = 3.47; cluster-level p_FWE_ = n.s.).

### Source reconstruction results

Source reconstruction of the instruction effect was performed for the interval between 122 and 180 ms, corresponding to the significant instruction scalp effect. Following IID source reconstruction, statistical parametric maps were thresholded at p_FWE_ < .05 at both cluster and peak-level. All MNI coordinates of significant effects are reported in Table 2. The inhibition vs. facilitation contrast (Fig. 5) revealed sources in right inferior frontal gyrus (peak-level T_max_ = 5.25; cluster-level p_FWE_ = .006) and right inferior temporal gyrus (peak-level T_max_ = 4.55; cluster-level p_FWE_ = .03). Instead, the facilitation vs. inhibition contrast (Fig. 5) was associated with sources in left insula (peak-level T_max_ = 8.24; cluster-level p_FWE_ = .001), left inferior frontal gyrus (peak-level T_max_ = 6.94; cluster-level p_FWE_ = .007), left middle frontal gyrus (peak-level T_max_ = 6.43; cluster-level p_FWE_ = .004), left supplementary motor area (peak-level T_max_ = 5.87; cluster-level p_FWE_ = .001), as well as bilateral precentral gyri (peak-level T_max_ = 5.48; cluster-level p_FWE_ = .005). Similar patterns of sources were identified for the contrasts inhibition vs. facilitation and inhibition vs. baseline, as well as for contrasts facilitation vs. inhibition and facilitation vs. baseline (Table 2). No significant effect was found for the two modulation conditions (inhibition and facilitation) vs. baseline.

### Exploratory analysis: identical stimuli perceived as painful vs. non-painful

Finally, we carried on an explorative analysis as suggested by a reviewer to elucidate potential ERP differences between subjective pain perception and objective physical intensity. We thus analyzed two additional contrasts at the scalp-level; i.e., low-intensity stimuli classified as non-painful vs. painful and high-intensity stimuli categorized as painful vs. non-painful, regardless the instruction conditions. We found that low-intensity stimuli judged as non-painful elicited increased positive amplitudes at 396–416 ms over occipito-parietal electrodes with respect to stimuli of identical physical intensity but classified as painful (i.e., peak-level T_max_ = 4.27; cluster-level p_FWE_ = .01). Further, uncorrected results suggested that high-intensity stimuli judged as painful vs. non-painful could evoke different neural activity at 26–52 ms and 90–104 ms; however these results were not significant after cluster correction. It is important to note that this analysis is underpowered given that lower amount of trials for the critical conditions (high-intensity stimuli perceived as non-painful and low-intensity stimuli perceived as painful).

## Discussion

The present study investigated the neural mechanisms by which mental imagery produces hypoalgesia and hyperalgesia in pain perception. Participants utilized mental images of a glove or a wounded forearm as strategies to attenuate or exacerbate their pain perception. We considered two main hypotheses to dissociate whether modulatory imagery effects were primarily driven by the redirection of attention away from upcoming sensations (a-directional hypothesis) or by altered processing of sensory and affective features embedded within each modulation condition (directional hypothesis). We found robust support for the latter; the participants' ability to modulate their subjective pain experience was paralleled by content-specific alterations of the signal discrimination performance, reaction times and the N2 pain-related component between 122 and 180 ms.

In accordance with the directional hypothesis, both inhibitory and facilitatory instructions moved the experience of pain intensity and unpleasantness towards imagined outcomes. When participants actively inhibited pain, not only did subjective pain perception decrease, but the detection of high-intensity stimuli as painful was also delayed. Moreover, high-intensity stimuli were more frequently categorized as non-painful and participants were less willing to report pain under inhibition. Congruently, when participants actively facilitated pain, the increase in perceived pain intensity and unpleasantness was associated with a delayed judgment of low-intensity stimuli as non-painful. Further, participants showed increased classification of low-intensity stimuli as painful and were more willing to report pain under facilitation. We also found evidence for changes in amplitudes of the pain-related N2 potentials by the content of instruction at 122–180 ms. However, no intensity by instruction interaction was observed in the time range of the N2, suggesting that the observed neural mechanisms underlying the modulation of pain are not pain-specific, but rather reflect modulatory mechanisms common to both pain and somatosensory-related processing (Bromm and Scharein, 1982). With respect to the N2 amplitudes, the two modulatory conditions diverged, with inhibition and facilitation respectively increasing or decreasing the magnitude of these negative potentials with respect to baseline. We note that given the larger N2 amplitudes for high vs. low stimulus intensity, one may have expected N2 amplitudes to decrease under inhibition vs. baseline (Bromm and Lorenz, 1998; Dowman, 1994). Surprisingly, here we observed the opposite effect; participants reported reduced pain under inhibition, but pain inhibition elicited greater N2 amplitudes. This finding contrasts with the interpretation of these potentials as objective pain intensity markers, suggesting that the magnitude of the N2 response cannot be considered a direct read-out of pain intensity (Bromm and Lorenz, 1998), but rather reflects a complex pain-related process integrating stimulus salience and cognitive expectations (e.g., Legrain et al., 2011). This pattern of N2 potentials fits with recent evidence that both sensory prediction errors conveyed by forward connections and top-down predictions conveyed by backward connections are implicated in the generation of late cortical ERPs (> 100 ms) (Garrido et al., 2007). For example, when a subject expects to have less pain (inhibitory mental imagery), but actually receive a painful stimulus, this would produce a net positive pain prediction error. In contrast, when a participant expects to feel more pain (facilitatory mental imagery), but actually receives less pain, the relative pain prediction error is negative. Thus, the directed alterations of the N2 potentials observed here may reflect the interaction between the sensory expectations encoded in the mental imagery and the actual sensory input. Future work would benefit from directly manipulating expectations to better determine the role of hypoalgesic and hyperalgesic prediction errors in influencing the amplitude of pain-related potentials.

Within the 122–180 ms time window corresponding to the N2 instruction effect, neural activity associated with inhibitory vs. facilitatory mental images was localized in the right inferior frontal and temporal regions (Fig. 5). These right-lateralized sources are consistent with evidence of right-hemisphere dominance in pain processing (Symonds et al., 2006) and inhibitory control (Garavan et al., 1999). Specifically, the right inferior frontal gyrus is part of a fronto-parietal network of brain regions involved in inhibitory control across a wide range of cognitive tasks (Hampshire et al., 2010). Further, previous studies showed that rIFG interacts with temporal brain regions during the retrieval of semantic information from long-term memory (Aron et al., 2004) and suppressing intrusive thoughts (Anderson et al., 2004). Our results are in agreement with the general role of right IFG in “reconfiguring a representation of a currently attended input” (Hampshire et al., 2010) and in generating inhibitory outputs. In the present experiment, right inferior frontal and temporal regions are likely involved in retrieving an alternative inhibitory content driven by the voluntarily generation of a mental image.

Instead, sources of facilitatory vs. inhibitory mental imagery were identified in left insular, inferior frontal, middle frontal, precentral and supplementary motor areas. Sources in the left hemisphere are consistent with the sensory stimulation of the right hand. In particular, these regions partially overlap with a network of cortical regions involved in salience processing and cognitive emotion regulation (Buhle et al., 2014; Kalisch, 2009; Kohn et al., 2014). Consistent with these findings, cognitive up-regulation and down-regulation of negative emotions have been shown to rely on both shared and distinct prefrontal activations. In particular, up-regulation (i.e., facilitation) has been uniquely linked to the recruitment of left medial prefrontal regions likely involved the retrieval of emotional contents; whereas down-regulation (i.e., inhibition) specifically recruited right lateral prefrontal regions involved in inhibitory cognitive control (Ochsner et al., 2004). Although these findings strongly support a directional effect, the precise regulatory mechanism underlying hypoalgesic and hyperalgesic mental imagery effects can be related to a variety of explanations; for instance, expectation, placebo–nocebo and cognitive reappraisal of negative affect.

### Pain-related mental imagery as expectation, placebo, or reappraisal?

The directional pain modulation driven by mental-imagery supported by our results might be differentially attributed to expectation, placebo, and reappraisal (Ochsner and Gross, 2005; Tracey, 2010; Wiech et al., 2008). We examine each interpretation in turn. First, the modulatory effects can be related both to expectations about pain itself and expectations about mental imagery. An expectation of reduced or increased pain is sufficient to alter how events are attended and thus experienced (Atlas et al., 2010; Keltner et al., 2006; Koyama and McHaffie, 2005). Alternatively, expectations embedded in mental imagery can be conceived as a placebo- or a nocebo-like effect. Indeed, imaginative suggestions include elements of both verbal suggestions and conditioning, by virtue of the associative content of a protective glove and excruciating wound with pain relief and pain exacerbation, respectively. Interestingly, placebo effects have been postulated to rely upon patients' mental imagery (Kojo, 1988). A final possibility is that mental imagery sustains reappraisal, which operates through cognitive reshaping of the stimulus meaning (Gross, 2001). In the context of pain, the perceived controllability of pain is thought to change the meaning of the upcoming stimulation (i.e., reappraisal), thereby reducing pain perception (Wiech et al., 2006, 2008). Expectation, placebo and reappraisal can be unified in terms of a common mechanism, in terms of integrating between bottom-up sensory signals and top-down predictions (e.g., Frith and Dolan, 1997). These differential interpretations are ripe for future experimental research into the underlying mechanisms driving the potent content-specific pain modulations observed here.

Our findings supporting the role of mental imagery in altering pain-related cognitive and emotional processing may shed light on the putative mechanisms underlying the effects of spontaneous mental images in patients suffering of chronic pain. For example, Philips (2011) showed that chronic pain patients frequently experience intrusive negative images linked to pain and one of the common theme of such images are physical and anatomical details of the pain or injury. Moreover, Berna et al. (2011, 2012) reported that some patients suffering chronic pelvic pain described not only negative imagery, but also coping images such as “imagery of treatment applied to the body” to reduce pain sensations. It is thus possible that the neurocognitive mechanisms observed in the present study are involved in spontaneous imagery reported in clinical settings.

### Limitations

A potential limitation of the present study is the lack of subjective ratings for non-painful stimuli. This limitation partially precludes us from determining whether the facilitation of non-painful stimuli corresponded to an allodynic effect at the perceptual level. A related worry is that pain ratings may have somehow biased responses to painful versus non-painful stimuli. While these issues should be raised in future studies, we note that the subjective effects were well mirrored behaviorally, with mental imagery consistently modulating reaction times, stimulus judgments and response biases. The consistency of our findings across behavioral, subjective, and electrophysiological measures, coupled with the fact that pain ratings were collected for “the most painful stimuli” across blocks of both painful and non-painful conditions thus suggests that these effects depended upon mental-imagery regulation rather than non-specific effects of ratings. However, future research may benefit from collecting ratings following individual stimulus presentations to better elucidate this issue.

## Conclusions

In conclusion, our results converge towards an interpretation of mental imagery as a flexible tool to alter pain and somatosensory sensations according to specific contextual expectations. We provided novel evidence for content-specific hypoalgesic and hyperalgesic imagery in modulating perception in opposite directions, biasing the perceptual boundary between high and low-intensity stimuli towards the instructed outcome, and modulating pain-related cortical responses at 122–180 ms. More specifically, inhibitory effects were associated with the recruitment of right lateralized brain regions commonly activated during cognitive control, whereas facilitatory pain effects were related to increased activity within salience and affective cortical regions. An important implication is that mental imagery may be a particularly relevant strategy for advancing clinical understanding and management of pain, such as in chronic pain and post-operative recovery, as well as further elucidating the neurocognitive mechanisms underlying pain regulation.

## Figures and Tables

**Table 1:** The significant clusters thresholded at p_FWE_ < .05 cluster-level and p_UNC_ < .001 peak-level for each instruction contrast. H < L = low-intensity over high-intensity stimuli, contrast-weight = [− 1 − 1 − 1 1 1 1]; H > L = high-intensity over low-intensity stimuli, contrast-weight = [1 1 1 − 1 − 1 − 1]; I > F = inhibition over facilitation, contrast-weight = [1 0 − 1 1 0 − 1]; I < F = facilitation over inhibition contrast-weight = [− 1 0 1 − 1 0 1]; I < B = baseline over inhibition, contrast-weight = [1 − 1 0 1 − 1 0].

Contrast	Effect	Cluster-level		Peak level		Peak coordinates		
P_FWE_	k_E_	P_UNC_	T	x (mm)	y (mm)	z (ms)
H < L	Main effect intensity Increased N2 for H vs. L	< 0.001	24,571	< 0.001	5.88	2	24	128
< 0.001 < 0.001 < 0.001 < 0.001	5.85 5.01 4.30 4.10	11 − 55 55 − 2	16 5 18 16	128 130 130 102
H > L	Main effect intensity Increased P2 for H vs. L	< 0.001	202,708	< 0.001 < 0.001 < 0.001 < 0.001 < 0.001 < 0.001 < 0.001 < 0.001 < 0.001 < 0.001 < 0.001 < 0.001 < 0.001 < 0.001 < 0.001 < 0.001	10.56 10.48 9.97 9.49 9.46 8.41 6.21 6.05 6.03 4.85 4.85 4.73 4.67 4.65 4.60 4.55	− 8 − 15 − 13 17 15 28 0 − 6 4 23 − 11 23 6 2 4 17	− 19 − 6 − 17 − 36 − 30 2 − 92 61 67 − 62 − 87 − 62 − 65 − 62 − 95 69	292 294 322 318 322 320 360 318 318 394 394 414 202 210 396 258
I < F	Main effect instruction Increased N2 for I vs. F	< 0.001	28,696	< 0.001 < 0.001 < 0.001 < 0.001 < 0.001 < 0.001 < 0.001 < 0.001 < 0.001 < 0.001 < 0.001 < 0.001 < 0.001 < 0.001	5.00 4.83 4.59 4.58 4.06 4.04 4.01 3.93 3.77 3.75 3.59 3.56 3.41 3.40	28 36 − 21 26 53 − 28 34 30 28 42 53 − 32 2 − 19	56 5 18 21 37 10 − 46 59 10 48 37 − 46 − 22 − 19	160 160 172 170 160 148 162 122 122 124 122 180 126 126
I < B	Main effect instruction Increased N2 for I vs. B	0.01	10,347	< 0.001 < 0.001 < 0.001 < 0.001 < 0.001 < 0.001 < 0.001 < 0.001	4.21 4.20 4.13 3.94 3.89 3.87 3.65 3.56	− 13 − 23 − 45 0 − 34 − 34 30 13	− 27 10 − 52 − 49 − 14 − 46 − 6 21	156 163 168 156 160 168 156 158

**Table 2:** The significant clusters were thresholded at p_FWE_ < .05 peak and cluster-levels, with a minimum cluster size of 10 voxels, for each instruction contrast. I > F = inhibition over facilitation, contrast-weight = [1 0 − 1 1 0 − 1]; F > I = facilitation over inhibition contrast-weight = [− 1 0 1 − 1 0 1]; I > B = inhibition over baseline, contrast-weight = [1 − 1 0 1 − 1 0]; F > B = facilitation over baseline, contrast-weight = [0 − 1 1 0 − 1 1]. Anatomical labels were based on cytoarchitectonic probabilistic maps; R = right, L = left.

Contrast	Area	Cluster-level		Peak level		Peak coordinates		
P_FWE_	N_VOXEL_	P_FWE_	T	x (mm)	y (mm)	z (mm)
I > F	R inferior frontal gyrus	0.006	115	0.003	5.25	42	32	16
0.004	5.18	42	34	22
R inferior temporal gyrus	0.030	13	0.037	4.55	54	− 60	2
0.039	4.54	56	− 56	4
F > I	L insula	0.001	246	0.001	8.24	− 38	6	10
0.001	5.68	− 48	− 12	16
L inferior frontal gyrus	0.007	95	0.001	6.94	− 42	6	20
L middle frontal gyrus	0.004	148	0.001	6.43	− 38	20	38
L supplementary motor area	0.009	78	0.001	5.87	− 6	4	50
0.001	5.61	− 8	10	52
0.001	5.48	− 8	14	50
L precentral gyrus	0.005	126	0.001	5.48	− 26	− 18	66
0.008	4.99	− 14	− 20	62
L middle frontal gyrus	0.018	38	0.003	5.24	− 20	36	34
L middle frontal gyrus	0.021	29	0.004	5.19	− 32	48	24
R precentral gyrus	0.021	29	0.012	4.88	10	− 26	70
L inferior frontal gyrus	0.030	13	0.014	4.83	− 40	28	− 6
I > B	R middle frontal gyrus	0.003	161	0.002	5.43	42	40	12
R inferior parietal lobe	0.012	60	0.002	5.34	58	− 54	24
R inferior frontal gyrus	0.017	41	0.007	5.01	48	16	16
R middle temporal gyrus	0.015	47	0.008	5.01	50	− 64	4
0.035	4.58	54	− 56	4
F > B	L insula	0.023	25	0.002	5.44	− 40	8	10
L insula	0.034	4.58	− 40	− 2	14
L inferior frontal gyrus	0.027	18	0.007	5.05	− 44	6	20
L paracentral lobule	0.028	16	0.007	5.04	− 6	− 40	74
R precentral gyrus	0.023	24	0.007	5.02	8	− 26	72
L precentral gyrus	0.017	40	0.011	4.92	− 24	− 22	60
